# Academic Performance of Students with the Highest and Mediocre School-leaving Grades: Does the Aptitude Test for Medical Studies (TMS) Balance Their Prognoses?

**DOI:** 10.3205/zma001006

**Published:** 2016-02-15

**Authors:** Guni Kadmon, Martina Kadmon

**Affiliations:** 1 Heidelberg University, Medical Faculty, Heidelberg, Germany; 2 Carl-von-Ossietzky-University of Oldenburg, Faculty of Medicine and Health Sciences, Oldenburg, Germany

**Keywords:** student admission, undergraduate medical training, aptitude, test for medical studies, TMS, school-leaving GPA

## Abstract

**Background:** Admission to undergraduate medical training in Germany occurs by central and local pathways. Central admission includes two distinct groups: Students with top school-leaving grades (best-SLG group) and students with inferior school-leaving grades who are admitted with a delay of up to seven years (delayed admission group). Students with academic difficulties and early dropouts are present in both groups. Local admission at our university involves the German Test for Medical Studies (TMS) and allows the admission by merit of students with a wide range of school-leaving grades.

**Aims: **To examine the justification of a TMS-based strategy to reduce the admission of potentially weak best school-leavers and enhance the admission of potentially able candidates with mediocre school-leaving grades.

**Method: **The prognostic contribution of the school-leaving (SL) GPA and the TMS to academic performance and to continuity in the pre-clinical part of the undergraduate medical program was examined in two study groups: best school leavers (SL grade 1.0, SL-GPA 823-900 points) and mediocre school leavers (SL grades 2.0-2.3, SL-GPA 689-660 points). The outcomes in both groups were compared in relation to their TMS results. The prospective study included four consecutive cohorts.

**Results:** In each study group the TMS predicted the academic performance (β=0.442-0.446) and the continuity of studies (OR=0.890-0.853) better than the SL-GPA (β=0.238-0.047; OR=1.009-0.998). Attrition was most strongly associated with failing to take the TMS (OR=0.230-0.380). Mediocre school leavers with TMS scores ≥125 performed as well as the best school leavers. Mediocre school leavers with TMS scores between 110-124 performed on average less well but within the required standards. Best school leavers with mediocre TMS scores and 30% of the best school leavers who hadn't taken the TMS performed less well than most mediocre school leavers with high TMS scores.

**Discussion:** The TMS appears to differentiate between potentially successful and less successful students in both GPA categories. Mediocre school leavers (SLG 2.0-2.3) with exceptionally high TMS results reach better pre-clinical examination results than best school leavers (SLG 1.0) with mediocre TMS results. Thus, the present data justify the use of the TMS to facilitate the participation of mediocre school leavers in the competition for admission slots.

## Zusammenfassung

**Hintergrund: **Die Auswahl Medizinstudierender wird in Deutschland teils zentral, teils lokal durchgeführt. Die zentrale Auswahl umfasst zwei Gruppen: Studierende mit den besten Hochschulzulassungsnoten (Abiturbeste) und Studierende mit schlechteren Abiturnoten, die erst nach einer Wartezeit von bis zu sieben Jahren nach Schulabschluss zum Studium zugelassen werden (Wartezeitquote). Studierende mit Lernschwierigkeiten sowie solche, die das Studium frühzeitig abbrechen, befinden sich in beiden Gruppen. Die hochschuleigene Studierendenauswahl (AdH) unserer Fakultät berücksichtigt das Ergebnis des Tests für Medizinische Studiengänge (TMS) und ermöglicht die leistungsbezogene Zulassung von Studierenden mit einer breiten Spannweite an Abiturnoten.

**Ziel: **Zu untersuchen, ob die Gewichtung des TMS in der hochschuleigenen Auswahl gerechtfertigt ist, die darauf abzielt, die Zulassung potentiell leistungsschwacher Abiturbesten zu reduzieren und stattdessen die Zulassung potentiell leistungsstarker Bewerber zu fördern, die mittelmäßige Schulabgangsnoten mitbringen.

**Methode: **Der prognostische Beitrag der Abiturpunktzahl und des TMS zur Studienleistung und zur Studienkontinuität im vorklinischen Abschnitt des Medizinstudiums wurde an zwei Studierendengruppen untersucht: Abiturbeste (Abiturnote 1,0, Abiturpunkte 823-900) und mittelmäßige Abiturienten (Abiturnoten 2,0-2,3, Abiturpunkte 689-660). Das Outcome beider Gruppen wurde im Verhältnis zu ihren TMS-Ergebnissen verglichen. Die Studie umfasste vier aufeinander folgende Kohorten.

**Ergebnisse:** In beiden Gruppen sagte der TMS die Studienleistung (β=0,442-0,446) und die Studienkontinuität (OR=0,890-0,853) besser vorher als die Abiturnote (β=0,238-0,047; OR=1,009-0,998). Die Studienverlängerungs- und -abbruchsrate war am stärksten mit der Nicht-Teilnahme am TMS assoziiert (OR=0,230-0,380). Studierende mit mittelmäßigen Abiturnoten und TMS-Ergebnissen (standardisiertem Testwert) ≥125 erzielten ähnlich gute Studienleistungen wie die Abiturbesten. Mittelmäßige Schulabgänger mit TMS-Ergebnissen zwischen 110-124 erbrachten im Schnitt weniger gute aber noch ausreichende Studienleistungen. Abiturbeste mit mittelmäßigen TMS-Ergebnissen und 30% der Abiturbesten, die nicht am TMS teilgenommen hatten, erreichten nicht das Studienleistungsniveau der meisten Studierenden mit mittelmäßigen Abiturnoten und sehr guten TMS-Leistungen.

**Diskussion: **Der TMS scheint, zwischen potenziell erfolgreichen und weniger erfolgreichen Studierenden beider Abiturnotenbereiche zu differenzieren. Mittelmäßige Abiturienten mit besonders hohen TMS-Ergebnissen erreichten bessere Studienergebnisse im vorklinischen Studienabschnitt als die Abiturbesten, die im TMS nur mittelmäßigen Erfolg hatten. Diese Beobachtungen rechtfertigen die Anwendung des TMS, um die Chancen von Studienbewerbern mit mittelmäßigen Abiturnoten am Wettbewerb um die Studienplätze zu erhöhen.

## Introduction

The academic performance at the secondary education or pre-university college level is generally considered as the strongest indicator for academic merit in tertiary education. The prognostic validity of school-leaving grades for academic performance in undergraduate medical training may vary between institutions and curricular years [1] but it often exceeds 0.3 and even 0.5. [2], [3], [4], [5]. Strong prognostic validity of school-leaving grades has also been documented for other academic courses [4], [6] as well as for non-academic professions [7]. For this reason, school-leaving grades are utilised worldwide as the major yardstick of merit in the admission or pre-selection to medical school.

In Germany, about 10% of the medical school admission slots are allocated to legally defined privileged applicants. Of the remaining admission slots up to 20% are centrally allocated to applicants with top school-leaving grades ("best-SLG group"). Another 20% are reserved for applicants with inferior school-leaving grades who are admitted centrally with a delay of up to seven years ("delayed admission group"). Approximately 50% of the medical students are locally admitted by the selection criteria of the individual university, whereby the school-leaving grade must by law carry substantial weight in the admission decision (local admission group). 

The dominant role of the school-leaving grade in the admission to medical school has in recent years increasingly become a matter of discussion. Admission by school-leaving grades tends to result in homogeneous student bodies [8], [9] and to discriminate against multiple groups of applicants including graduates from public schools, applicants from socioeconomic and educationally deprived backgrounds [10], [11] and male school leavers [12], [13]. Moreover, school-leaving grades do not necessarily reflect non-cognitive qualities that are important for patient-centred medical practice. 

A broad spectrum of additional, cognitive as well as non-cognitive admission instruments has been developed to accommodate for the shortcomings of the school-leaving grades as an admission instrument. The cognitive admission instruments include general intelligence tests such as the SAT in the USA [14], SweSAT in Sweden [15], and the psychometric test in Israel [16], scientific knowledge tests such as the BMAT in Britain [17] and the Ham-Nat in Germany [18], and special aptitude tests for medical studies such as the MCAT in the USA [19], the UMAT in Australia [20], UKAT in Britain [21], and the Aptitude Test for Medical Studies (TMS) in Germany [22], [23] and Switzerland (EMS, [24]). Non-cognitive instruments including interviews [5], [25], [26], motivational essays [5], personal qualities assessment (PQA) [27], [28], and psychological tests [29] are also employed but generally lack reliable prognostic values for performance in medical school. However, instruments that depict a limited spectrum of non-cognitive traits may be of prognostic relevance with respect to specific competencies, examination types, or course formats [30], [31]. 

In view of the prognostic insecurity of non-cognitive admission instruments Brown and Lilford [32] recommended limiting student selection to cognitive instruments. Conceivably, applicants with different school-leaving grades reflecting different school histories may demonstrate similar aptitude for medical studies by alternative cognitive admission instruments. Such applicant cohorts may represent a wider range of interests and competencies than applicants with a uniform school-leaving grade. On the basis of this rationale our faculty developed a compensatory admission procedure that is primarily based on the school-leaving GPA and the TMS score. To this end a ranking formula was used that is given in the methods section.

The TMS is composed and administered once yearly by ITB Consulting^®^ LTD. It comprises nine parts, takes approximately 5 hours and 10 minutes, and does not require prior academic knowledge. In a slightly different form it is also used in Switzerland (EMS, [24], [33]). Four parts test scientific and mathematical thinking as well as text and data comprehension. The scores achieved in these parts correlate moderately (r=0.33) with the school-leaving GPA [22]. The remaining five parts test visual cognition, pattern analysis, graphic and verbal memory. These parts do not substantially correlate with the school-leaving GPA (r=0.16) [22]. The scores achieved in the test are standardised each year with mean 100 and standard deviation 10. Preparation for the test is beneficial to the result [34], suggesting that it might also reflect motivation. 

The advantage of assessing applicants who are weak in one instrument also by an alternative instrument which may better reflect their aptitude has been discussed [35], [36]. The compensatory ranking formula enables potentially able applicants to compensate for poor school-leaving grades by strength in the TMS as an additional measure of merit. 

In an earlier study [2] it has been observed that some students of the best-SLG group have academic difficulties in undergraduate medical training whereas some students with mediocre school-leaving grades reach high levels of performance. However, students with mediocre school-leaving grades normally are admitted to medical school with several years’ delay and tend to withdraw from the course pre-maturely due to age-related factors. These observations are supported by the experience that the introduction of the Medical College Admission (Moss) Test (MCAT) in the USA resulted in improved prediction of academic performance, drastically reduced attrition [37], and increased diversity of students by race and religion [38]; the BioMedical Admissions Test (BMAT) better predicts academic success of candidates from low ranking schools than personal statements [39], and veterinary students may become good veterinary doctors despite having inferior school leaving grades [40]. We therefore suggested that potentially able applicants with mediocre school-leaving grades should be given the chance to compete for study places within the regular admission procedure at the expense of applicants with top school-leaving grades but lower potential for academic success [2]. The present work aims at examining whether the compensatory admission procedure described above can identify best school-leavers with low potential and mediocre school-leavers with high potential for success in the undergraduate medical program. To this end the academic performance and continuity of the students of the central admission groups, the best-SLG group and the delayed admission group, were compared to the performance and continuity of the locally admitted students with the corresponding school-leaving grades. The differential predictive values of the school-leaving grade and the TMS for academic performance and continuity of studies were statistically elucidated. The work focused in three consecutive studies on the following questions:

- Study 1: Does the TMS help to predict the differential academic performance and continuity of students that have the best school-leaving grade 1.0 (best school leavers)?
- Study 2: Does the TMS help to predict the differential academic performance and continuity of students whose school-leaving grades are on the level of the national average (2.0-2.3 – mediocre school-leavers)?
- Study 3: Do students with mediocre school-leaving grades but high TMS scores reach levels of academic performance that justify their inclusion in the primary competition for admission slots?

## Methods

### Participants and inclusion criteria

The participants were medical students of the Heidelberg Medical Faculty of Heidelberg University who enrolled in 2009, 2010, 2011, and 2012. Included were:

- All students with grade 1.0 in the German school-leaving certificate (“Abitur”).
- All students having German school-leaving grades 2.0-2.3.

#### Exclusion criteria

Excluded from the study were students with foreign school-leaving certificates and students admitted by different pathways than the regular admission procedure (priority admissions, cases of hardship, second-degree students, admissions by lawsuit). Students who hadn't taken the TMS were excluded from analyses involving the TMS score. They were included in comparisons between the students who had taken the TMS and those who hadn't taken it.

#### Data recruitment and data protection

The study was performed in connection with the quality management of the admission procedure of the Heidelberg Medical Faculty. Birth dates, registration and de-registration dates, examination results, and the date of passing the first part of the Medical Licensing Examination (M1) were retrieved from the data bank of the faculty. The school-leaving grades of the locally admitted students were retrieved from their application files. The school-leaving grades of the centrally admitted students were reported by the Foundation for Higher Education Admissions (SfH). The data were tabulated in MS Excel^®^ and anonymised prior to their analysis by removal of the columns that contained personal data except for age and gender. The work was approved by the ethics committee of the medical faculty (file #S-440/2009).

#### Assessment scales

*School-leaving-GPA:* It ranges from 240 points (pass) to 840 points or 300 (pass) to 900 points. The 840 point scale was converted to the 900 point scale when used. The two scales have been used in different years and are still differently used by different German federal states. 

*School-leaving grade:* The school-leaving GPA is officially converted to the school-leaving grade on the scale of 1.0 - 6.0 (1.0=best, 4.0=pass). The GPA scores corresponding to Grades 1.0, 2.0-2.3 are given in Table 1 (Tab. 1). 

*Standardised TMS score: *0-135 points, mean=100 points, SD=10 points. The TMS scores were divided into seven categories with the following score ranges: (1) ≥125, (2) 120-124, (3) 117-119, (4) 114-116, (5) 110-113, (6) 106-109, (7) ≤105. Wider TMS score intervals were chosen for the marginal categories (1,2,6,7) in ordert to increase the sample sizes of rare cases. Rare cases were included in the analysis since they were in the focus of interest with respect to the research questions. An eighth category was defined as the absence of TMS results. 

*Weighted compensatory ranking formula*: The compensatory local admission procedure was based on the following ranking formula, whereby only better than average TMS scores were considered: 


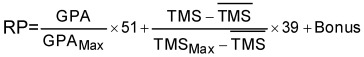


where RP=ranking points, GPA=school-leaving GPA, GPA_Max_=840 or 900 points depending on the respective GPA scale, TMS=standardised TMS score, 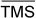
=mean standardised TMS score (100), TMS_Max_=130, Bonus=maximally 10 percent points for additional criteria. Since only few students qualified for bonus points the bonus was neglected in the present study.

*Faculty examination results: *The examinations included the chemistry exam of the first semester, the physics, human genetics, and the integrated exams (anatomy, physiology and biochemistry) of the second, third, and fourth semesters. Different point scales were transformed to the percent scale (100%=highest possible score in a given examination, 60%=pass). The arithmetic mean of the transformed pre-clinical examination results was defined as the mean pre-clinical grade and regarded as a measure for academic performance. 

The analysis of the students’ performance was based on the results of their first try at each examination. It was assumed that the first try better reflects the actual learning performance than the repetition of failed examinations. Passing the examinations is an obligatory condition for taking the M1 examination, but the time point for taking some examinations is flexible. 

*Continuity of studies *was analysed as passing the first part of the Medical Examination (M1) in the prescribed time of four semesters (regular continuity), at a later stage (prolonged studies), or withdrawing from the program prior to the M1 examination (dropout). 

#### Admission criteria

The admission criteria for the different admission groups were:

- Central admission:

1. *SLG-best group* – School-leaving grade (almost exclusively grade 1.0).
2. *Delayed admission group* – waiting time by number of semesters. (Applicants who do not comply with the regular admission criteria but are entitled to a study place by the constitutional freedom of occupational choice. They are admitted with a delay of up to seven years after leaving school.)
3. *Local admission* – school-leaving GPA with pre-selection cut-off at GPA 589 (grade 2.3), above-average TMS score (>100) and additional “bonus criteria” (vocational training and professional experience in medicine-related fields, success in education-related competitions, and voluntary social service). The applicants were ranked by the formula given above.

#### Study design

**Figure 1:**
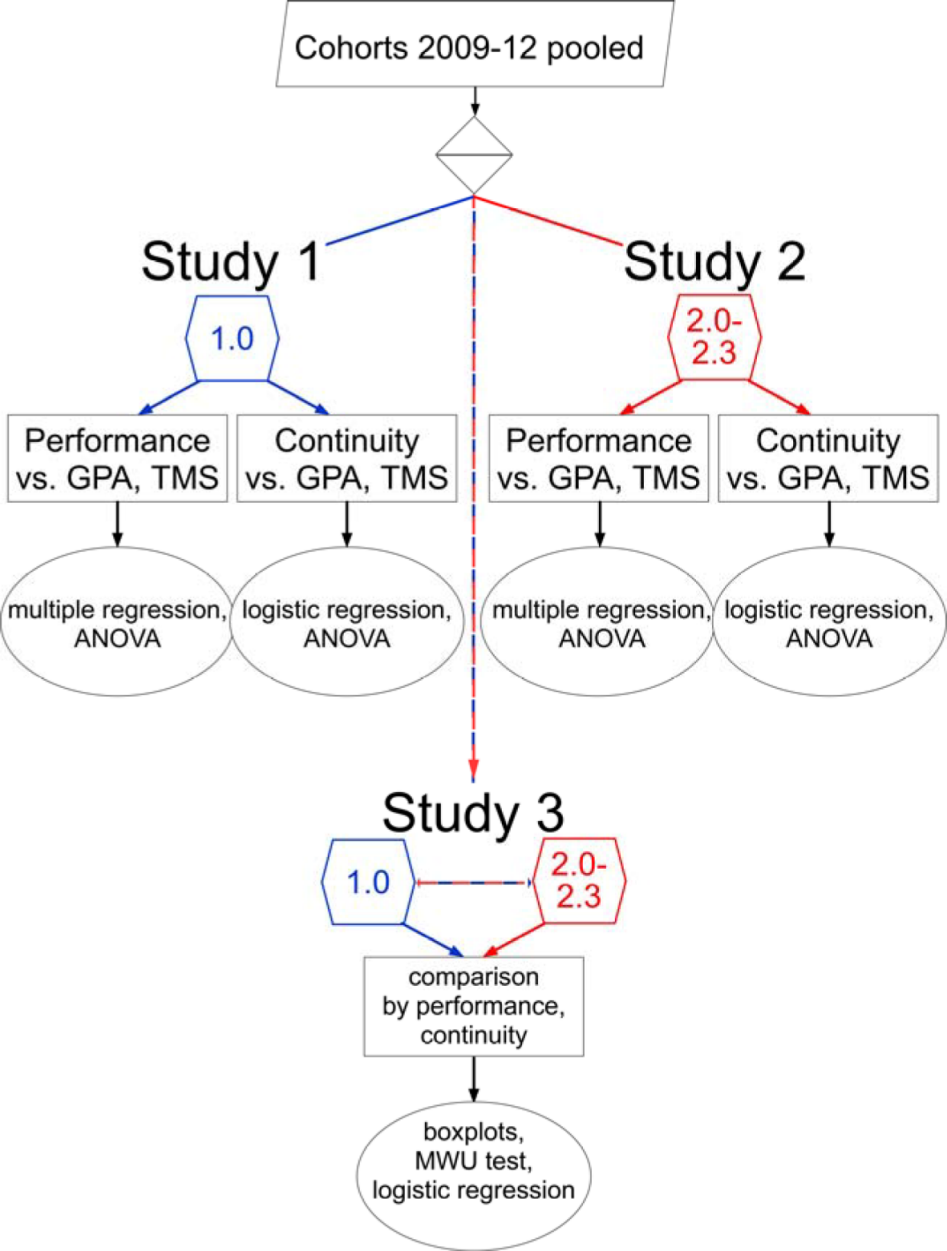
*Flow chart of the study design.* Study 1 and study 2 examine the academic performance and the continuity of studies of the students with the school-leaving grades 1.0 and 2.0-2.3, respectively, in the pre-clinical part of the medical course. The performance and the continuity of both groups are compared in the third study.

The study design is summarised in Figure 1 (Fig. 1) and table 1 (Tab. 1). The investigation included two study groups: students with the school-leaving grade 1.0 (best school leavers) and students with the school-leaving grades 2.0-2.3 (mediocre school leavers). For each study group the data of the students that had matriculated between 2009 and 2012 by the different admission procedures were pooled. The data included the school-leaving GPA, the TMS score, the faculty examination results and the continuity of studies.

The investigation was divided in three studies. Study 1 included the best school leavers and study 2 included the mediocre school leavers. In each of these two studies the relationship between the mean pre-clinical grades and the predictors school-leaving GPA and TMS score as well as between continuity of studies and these predictors was analysed. Additionally, the relationship between the outcome variables (mean pre-clinical grade, continuity of studies) and taking or not taking the TMS was also analysed. In study 3 the outcome variables of the two study groups were compared with respect to the given predictors. The study groups were examined in toto as well as in subgroups by TMS categories as defined above. 

#### Statistical methods

The predictive values of the school-leaving grade and of the TMS score for academic performance were examined by multiple linear regression as well as by ANOVA by TMS categories. ANOVAs were followed post-hoc by Bonferroni or non-parametric (Mann-Whitney U) tests. The predictive values of the school-leaving grade and the TMS score for continuity was examined by logistic regression. The possible risk for academic performance and for continuity by not taking the TMS was also examined by logistic regression. The mean TMS scores by continuity of studies were compared by ANOVA. In study 3 the academic performance of the two groups was compared in relation to their TMS results. To this end the TMS results were categorised as described above. The proportions of students of the two study groups who completed the pre-clinical part of the program in the prescribed time, after a delay, or dropped out were compared by χ^2^ test for proportions. The proportion of students who had taken (or not taken) the TMS was compared among the subgroups by continuity (regular continuity, prolonged studies, dropout) using the z test for proportions.

Basic statistics, distribution analyses, multiple linear and logistic regression analyses, Pearson correlations, confidence interval determinations, ANOVA, Kruskal-Wallis H test, Mann-Whitney U test, χ^2^ test, z-test for proportions and Boxplots were performed in IBM SPSS^®^ 21. Holm-Bonferroni correction for multiple comparisons was performed on an Excel^®^ template by Justin Gaetano [https://www.researchgate.net/publication/236969037_Holm-Bonferroni_Sequential_Correction_An_EXCEL_Calculator]. Participants with missing data were omitted from the respective analysis. SPSS output was exported to MS Excel^®^. Graphics were generated in Excel and finished in Canvas^®^ 10 (ACD Systems). 

## Results

### Study 1: Best school leavers (school-leaving grade 1.0)

#### Dependence of academic performance on GPA and TMS score

By multiple linear regression analysis both the school-leaving GPA and the TMS score contribute to the prediction of the mean pre-clinical examination result of the best school leavers. However, the predictive contribution of the school-leaving GPA (β=0.238) was weaker than the predictive value of the TMS (β=0.442; see Table 2 (Tab. 2), Point A). Sub-grouping the best school leavers by their TMS scores revealed two outstanding subgroups with respect to their academic performance (see Table 2 (Tab. 2), Points B and C). The one subgroup included students with TMS scores ≥125. TMS scores ≥125 are at least 2.5 standard deviations better than the mean score of all TMS participants. These students reached mean pre-clinical examination results that were significantly or nearly significantly better than examination results of the subgroups with lower TMS scores. The other subgroup included the students with TMS scores ≤105, that is, around the mean score of all TMS participants. Their mean pre-clinical examination results were significantly worse than those of most other subgroups. The students who had not taken the TMS also reached on average relatively low examination results (see Table 2 (Tab. 2), Point B). The mean pre-clinical examination results of the remaining subgroups with TMS scores between 106 and 124 did not differ significantly. 

##### Dependence of continuity of studies on GPA and TMS score

By logistic regression, the best school leavers' risk of prolonging their pre-clinical studies and the risk of dropping out of the course were not related to the school-leaving GPA (see Table 3 (Tab. 3), Point A). In contrast, lower TMS scores significantly predicted the prolongation of the studies albeit with an odds ratio of only 0.89 (see Table 3 (Tab. 3), Point A; OR<1 denotes the chance of not prolonging the studies). The odds ratio was possibly weak due to the small number of students who had taken the TMS and withdrew from the program. The students who prolonged their studies had significantly lower TMS scores than students who completed the pre-clinical part of the program in the prescribed time (see Table 3 (Tab. 3), Point C). 

A higher risk for prolonging the studies and for dropout was detected among the best school-leavers who had not taken the TMS as compared to those who had taken the TMS (OR 0.324 and 0.451, respectively; see Table 3 (Tab. 3), Point B). The majority (82 out of 97) of the best school leavers who had not taken the TMS belonged to the centrally admitted SLG-Best group. Thirteen had been admitted otherwise and were older at enrolment. 

#### Study 2: Mediocre school leavers (school-leaving grades 2.0-2.3)

##### Dependence of academic performance on GPA and TMS score

Multiple linear regression analysis did not reveal an association between the school-leaving GPAs of the mediocre school leavers and their mean pre-clinical examination grades (β=0.047, ns). In contrast, their TMS scores appeared to predict their pre-clinical performance similarly to the group of the best school leavers (β=0.446; see Table 4 (Tab. 4), Point A). The GPA ranges of both groups were similar (see Table 1 (Tab. 1)), whereas the range of the TMS scores of most mediocre school leavers was more restricted – 74% were within 8 score points, 117-124 (see Table 4 (Tab. 4), Point B). Therefore, their regression was possibly more strongly susceptible to outliers. 

Upon sub-grouping the mediocre school leavers by TMS scores a downward gradient of academic performance with decreasing TMS scores appeared as a general trend (see Table 4 (Tab. 4), Point B). However, the differences between the mean pre-clinical grades of the different subgroups were with the exceptions described below not significant (see Table 4 (Tab. 4), Point C), possibly due to the small size of most subgroups. 

The mean pre-clinical grades of the subgroups with TMS scores ≤105 and with no TMS result were significantly lower than in most other subgroups and were close to the "pass" mark of 60% (see Table 4 (Tab. 4), Points B and C). This outcome is more difficult to relate to the gradient in TMS scores than in the group of the best school leavers, since the mediocre school leavers with TMS scores <113 or no TMS belonged to the delayed admission group. They were several years older at enrolment than the rest of the students (see Table 1 (Tab. 1)).

##### Dependence of the continuity of studies on GPA and TMS score

The statistical relationship between the continuity of studies of the mediocre school leavers and their school-leaving GPAs and TMS scores was similar to the one observed in the group of the best school leavers. By logistic regression dropout was not related to the TMS score whereas the risk of study prolongation by low TMS scores was determined at an odds ratio of 0.85 (see Table 5 (Tab. 5), Point A). Also, the TMS scores of the mediocre school leavers who prolonged their studies were much lower than those of the mediocre school leavers who did not prolong their studies or withdrew from the program (see Table 5 (Tab. 5), Point C). Similarly as observed among the best school leavers, the highest risk for prolonging the studies and for dropout was associated with not having taken the TMS (see Table 5 (Tab. 5), Point B). As pointed out above, the interpretation of these observations is limited by the age difference between the mediocre school leavers with TMS scores <113 or no TMS and the mediocre school leavers with better TMS scores.

#### Study 3: Mediocre vs. best school leavers 

##### Academic performance by TMS categories

**Figure 2:**
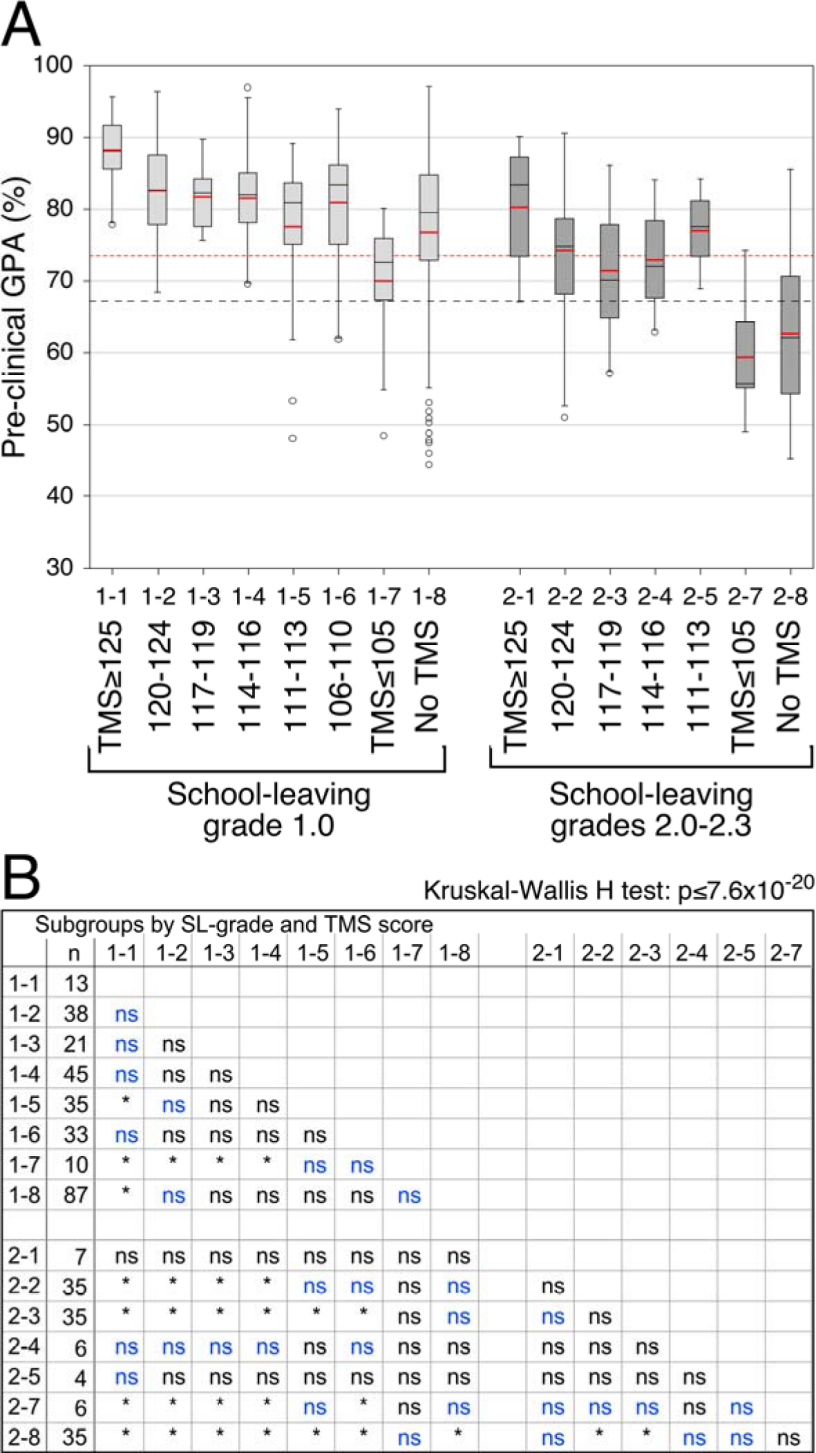
*Academic performance by school-leaving grade and TMS score.* A. Boxplots by TMS categories. The subgroups are denoted by a group number (1 or 2) and a subgroup number (1 to 8). The mean values are superimposed in red. Red and black broken lines: Mean and 25 percentile, respectively, of the performance of the mediocre school leavers with TMS>105. B. Kruskal-Wallis H test with pairwise post-hoc comparisons by Mann-Whitney U test with global α=0.05 and Holm-Bonferroni correction for multiple comparisons. *Significant at α=0.05; (n) number of cases; (ns) not significant; (blue print) significant prior to the Holm-Bonferroni correction.

Generally, the mediocre school leavers tended to perform less well than the best school leavers who had taken the TMS (see Figure 2 (Fig. 2), Point A). The differences were only partly significant (see Figure 2 (Fig. 2), Point B), possibly due to the small size of most subgroups of the mediocre school leavers. In both study groups the students with exceptionally high TMS scores (≥125) performed better than the rest of their respective group members and the students with mediocre TMS scores (≤105) performed less well than the rest of their respective group (see Figure 2 (Fig. 2), Points A and B). The academic performance of the best school leavers with TMS scores between 106 and 124 was on average relatively uniform at a level of mean pre-clinical grades around 82% (see Figure 2 (Fig. 2), Point A). A similar level of academic performance was achieved by the mediocre school leavers with TMS scores ≥125. Their pre-clinical grades did not differ significantly from those of the best school leavers including those with TMS scores ≥125. However, due to the small size of the subgroups with TMS scores ≥125 true differences may have been masked by type II error. The mediocre school leavers with TMS scores between 117 and 124 performed, on average, significantly less well than the majority of the best school leavers who had taken the TMS (see Figure 2 (Fig. 2), Points A and B). Yet, they performed clearly better than the minimal required standard of 60% ("pass"). Mediocre school leavers with TMS scores below 117 were too rare for drawing clear conclusions about them. 

The academic performance of the mediocre school leavers in the pre-clinical examinations was also compared to that of the best school leavers who had not taken the TMS. The differences were not significant. This finding is especially meaningful with respect to the mediocre school leavers with TMS scores ≥117 whose subgroups were large enough for reliable comparisons (see Figure 2 (Fig. 2), Points A and B). In fact, the mean performance of 27 best school leavers without TMS (31%) was lower in the pre-clinical examinations than the mean performance of the mediocre school leavers with TMS scores >105. Furthermore, 18 of the former (20%) performed less well than the 25 percentile of the latter (see Figure 2 (Fig. 2), Point A). Among the best school leavers with TMS scores ≤116 24% performed less well than the mean and 9% performed less well than the 25 percentile of the mediocre school leavers with TMS scores >105 (see Figure 2 (Fig. 2), Point A).

#### Continuity of studies

**Figure 3:**
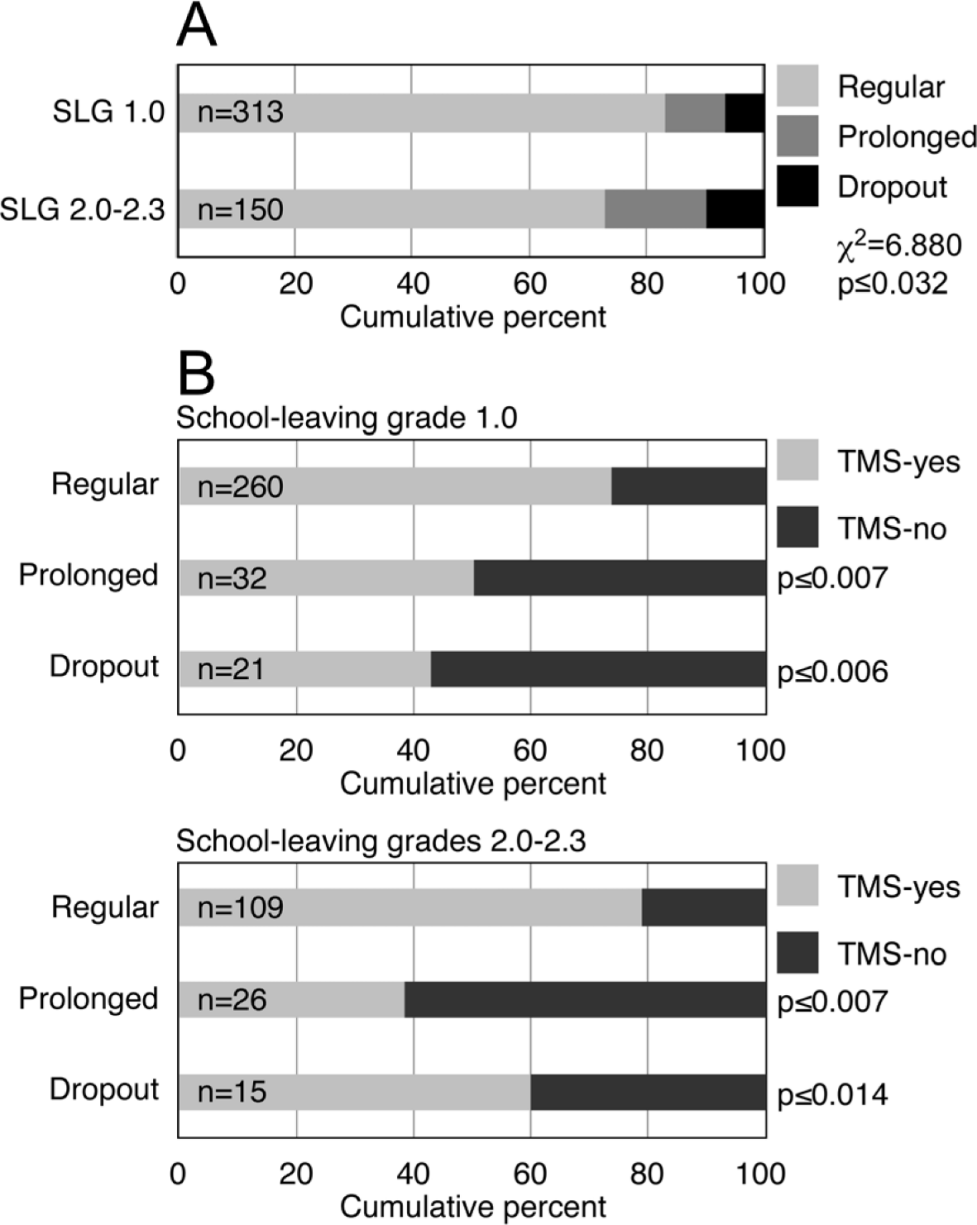
*Continuity of studies of best and mediocre school leavers*. A. Proportional distribution of the students who completed the pre-clinical program in the prescribed time (Regular) or later (Prolonged) or who withdrew from the program (Dropout) in the two study groups. (SLG) School-leaving grade. The distributions of both study groups were compared by χ^2^ test. B. Proportional distribution of the students who had taken the TMS (TMS-yes) or had not taken it (TMS-no) among the students who completed the pre-clinical program in the regular time, prolonged their studies or who dropped out. (p) The proportions of the students who had not taken the TMS among those who had prolonged their studies or dropped out were compared by z-test for proportions to the equivalent proportion among the students who had completed their pre-clinical studies in the regular time.

Similar proportions of students of both study groups, the best and the mediocre school leavers, completed the pre-clinical program. However, approximately 10% more best school leavers (83.1%) than mediocre school leavers (72.7%) completed it in the prescribed time (see Figure 3 (Fig. 3), Point A). In both groups the majority of students who completed the pre-medical program in the prescribed time had taken the TMS. Conversely, 40-60% of the students who prolonged their studies or dropped out had not taken the TMS (see Figure 3 (Fig. 3), Point B). 

The two study groups, the best and the mediocre school leavers, were pooled together for further analysis. The range of the school-leaving GPAs was larger in the pooled sample than in each study group alone. Yet, logistic regression indicated that also in the pooled sample the school-leaving GPA only marginally affected the prolongation of the studies or the withdrawal from the program if at all (see Table 6 (Tab. 6)). The TMS scores (see Table 6 (Tab. 6), Point A) and not having taken the TMS (see Table 6 (Tab. 6), Point B) represented similar risk potentials for attrition as separately observed in each study group (compare to Table 3 (Tab. 3) and table 5 (Tab. 5)). 

## Discussion

The present study explores the possibility of having excellent students who had left school with poor grades and having unsuccessful students who had excelled in secondary education. Clearly, it would be desirable to identify the applicants who occupy these "tails of the probability distribution" already during the admission process. It can be expected that students with varying school histories may contribute substantially to the diversity of the student body [41]. To accomplish this, a selection instrument must be employed that can detect potentially unsuccessful best school leavers and potentially successful mediocre school leavers. The ability of the TMS to fulfil this function was examined in the present work. 

### Prognostic values of the school leaving GPA and the TMS for academic performance and continuity of best and mediocre school leavers (studies 1 & 2)

*Academic performance:* Differences in academic performance were weakly related to GPA differences among the best school leavers and unrelated to the GPA among the mediocre school leavers. Moreover, attrition – prolongation of the studies and dropout – was also unrelated to the school leaving GPA. The range of the school-leaving GPA was similar in both study groups, just above 70 points. This range comprises only a fraction of the complete scale of GPA scores. Thus, the observed absence of a (clear) relationship between the outcome variables and the school-leaving GPA may be restricted to the study groups and cannot be generalised at this stage. 

The TMS, on the other hand, predicted the academic performance in both study groups with β values above 0.4. Upon closer inspection, in each group the mean academic performance of students with TMS results between one and 2.5 standard deviations above the mean score of all TMS participants was relatively uniform. The best school leavers having TMS scores within this range reached on average better examination grades than the corresponding mediocre school leavers. Nevertheless, the examination grades of the latter subgroups were on average also well above 60%, the required "pass" level. 

Exceptions to this general trend were observed in the marginal subgroups having either exceptionally high (better than 2.5 SD above the mean) or mediocre TMS results. In each study group the subgroup with exceptionally high TMS scores reached better mean examination grades than the remaining subgroups. This was most conspicuous in the group of the mediocre school leavers, although due to the small size of this subgroup the observed advantage was not statistically significant. The subgroups with TMS scores around 100, the mean of all TMS participants, performed significantly less well in the pre-clinical examinations than their fellow students with better TMS results. This observation is especially meaningful with respect to the best school leavers, since their subgroups did not differ in age. In the group of the mediocre school leavers, age is a latent variable that must be considered in the interpretation of the results. 

*Continuity of studies:* In both study groups the TMS was also a predictor of study prolongation. This result can be expected, since the prolongation of the pre-clinical period is often associated with academic difficulties [2]. Interestingly, the binary variable, "taking/not taking" the TMS, emerged in both study groups as the strongest predictor for both study prolongation and dropout. Therefore, future investigations should examine whether taking the TMS reflects motivation and identification with the chosen course of studies, and whether failing to take it might be associated with reduced motivation and endurance. 

#### Mediocre vs. best school leavers (study 3)

The comparison of the two groups revealed three relevant phenomena:

1. The mediocre school leavers with exceptionally high TMS scores performed in the pre-clinical examinations on average similarly well as the best school leavers with high TMS scores.
2. Quite a few best school leavers without TMS participation and with TMS results below 117 performed less well than many mediocre school leavers.
3. Differences in GPA could not explain the attrition even upon pooling the school-leaving GPAs of both study groups, whereas the TMS score did predict the attrition.

The prognostic risk for attrition was associated most strongly with not having taken the TMS.

The results indicate that the admission to medical school of mediocre school leavers with exceptionally high TMS results is fully justified. Moreover, it is questionable whether the *a priori* exclusion of such applicants from the primary competition for admission slots by faculties that do not employ compensatory admission instruments would be appropriate. The present findings also indicate that even the admission of mediocre school leavers with better TMS scores than one standard deviation above the mean of all TMS participants is more reasonable than the admission of best school leavers with mediocre TMS results. Our experience is that they are likely to enrich the diversity of the student body [41] and level out academically with the best school leavers in the clinical part of the program [2]. 

Taken together, the present findings confirm the need for public discussion about the obligatory attribution of priority weight to the school-leaving grade in the admission decision as required by German law. 

#### Advantage of voluntary admission tests

The assumption that taking the TMS may reflect motivation, identification, and endurance must await future verification. However, the current observations appear to support the importance of offering the TMS and perhaps any comparable admission test on a voluntary basis as an indicator of potential continuity and attrition. 

#### TMS credits in other German universities

The majority of the German medical faculties that acknowledge the TMS result in their admission procedure consider the TMS on an additive rather than a compensatory basis. They attribute a uniform credit to either the TMS equivalent grade 1.0 or to TMS percentile rank values ≥90. The TMS equivalent grade 1.0 includes in different years all TMS scores above 116 or above 117. The percentile rank value 90 encompasses the TMS scores above approximately 112. Consequently, exceptionally high TMS scores have no advantage, success in the TMS has a relatively small incremental effect on the admission decision, and applicants with school-leaving grades above approximately 1.8 are excluded from the competition for study places [42]. 

#### Demographic considerations

Demographic data of the students except for age and gender were not documented in the data bank of the faculty due to the data protection regulations as they are not relevant to the admission procedure. The age distribution of the students of the different admission groups has been published [2]. So far, we have not detected gender differences in performance and attrition in our faculty [2], and only minor gender differences in course evaluation have been found [43]. For these reasons and in order to avoid further sample fragmentation analysis by gender has not been performed in the present study.

#### Limitations of the study

The interpretation of the results is limited by a number of confounders as listed below. Most notably, the small size of the marginal subgroups of interest, the best school leavers with mediocre TMS scores and the mediocre school leavers with exceptionally high TMS scores, restricts the confidence in the generalizability of the findings. The association between the school-leaving grades and the age at matriculation as well as between taking or not taking the TMS and age may further limits the interpretation of the findings. The large number of possible examination dates available to the students renders the analysis of the sources of variance in a nested set-up impractical. The results must, therefore, be regarded as observations that pose questions to further generalising work, especially concerning the marginal subgroups.

#### Confounders and sources of bias

- Age: Due to the delayed admission procedure the school-leaving GPA scale and, partly, taking or not taking the TMS are linked to the age at enrolment. Age is a strong predictor for dropout, less so for academic performance [2].
- Variance restriction: The analyses focused on groups of the student cohorts that represent selected segments of the school-leaving GPA scale. They were further divided by TMS categories into subgroups that in part were very small. Consequently, the interpretation of the findings may be limited by variance restriction.
- Statistical errors: Due to variance restriction type I and type II errors possibly limit the reliability of the results.
- Examination results: Latent factors affecting academic performance such as health condition, personal and familial difficulties, exam anxiety, variations in examination severity, had not been documented and were neglected in the analysis.

## Conclusions

Advantage in admission should ideally be given to applicants who are likely to succeed in the course over those who potentially would fail to reach the desired level of performance regardless of their respective school histories. Moreover, it makes little sense to curtail the coping potential of potentially successful, consistent students by deferring their admission by several years only because top grades were not their top priority at high school. The present data indicate that a compensatory admission procedure involving the TMS should be considered for balancing the required substantial weight of the school-leaving grade in the assessment of potential academic aptitude.

## Acknowledgements

The authors wish to thank Martina Damaschke and Dr. Ariunaa Batsaikhan for their patient assistance in retrieving examination results and tabulating the data. They are also grateful to Dr. Janine Kahmann, Anna Kirchner, and Stefan Teichert for their advice on the admission procedures and their constructive discussions. Special thanks are due to Melanie Fröhlich for critical reading of the manuscript and her help in avoiding unnecessary mistakes.

## Competing interests

The authors declare that they have no competing interests.

## Figures and Tables

**Table 1:**
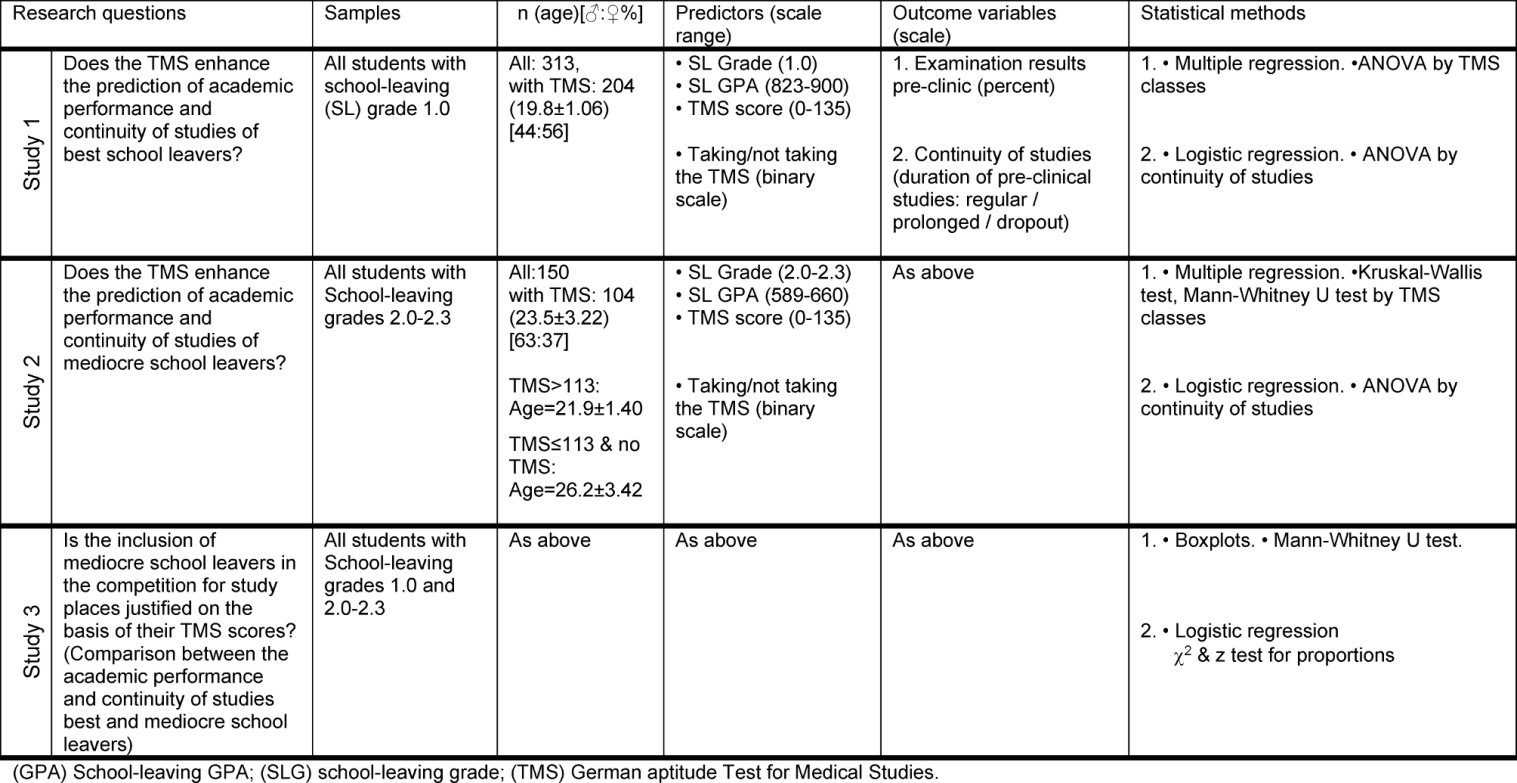
Study design

**Table 2:**
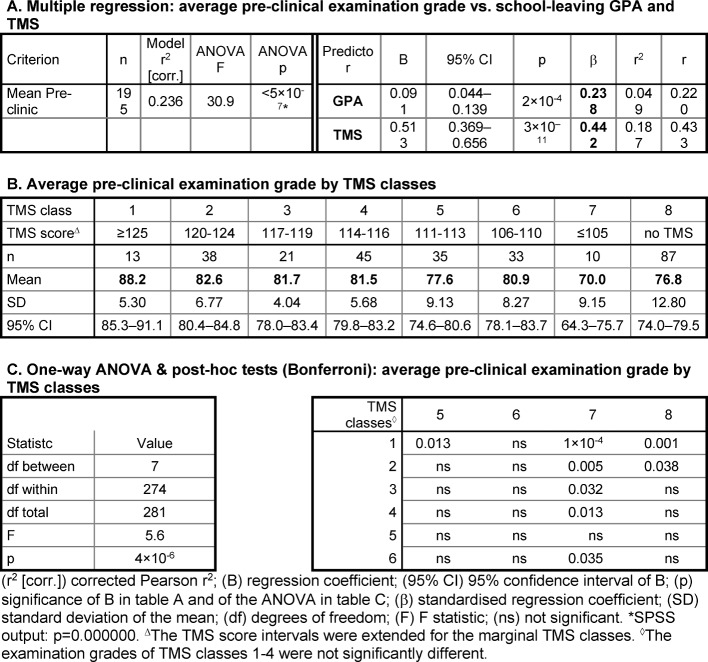
Relationship between academic performance, school-leaving GPA, and TMS score among students with the school-leaving grade 1.0

**Table 3:**
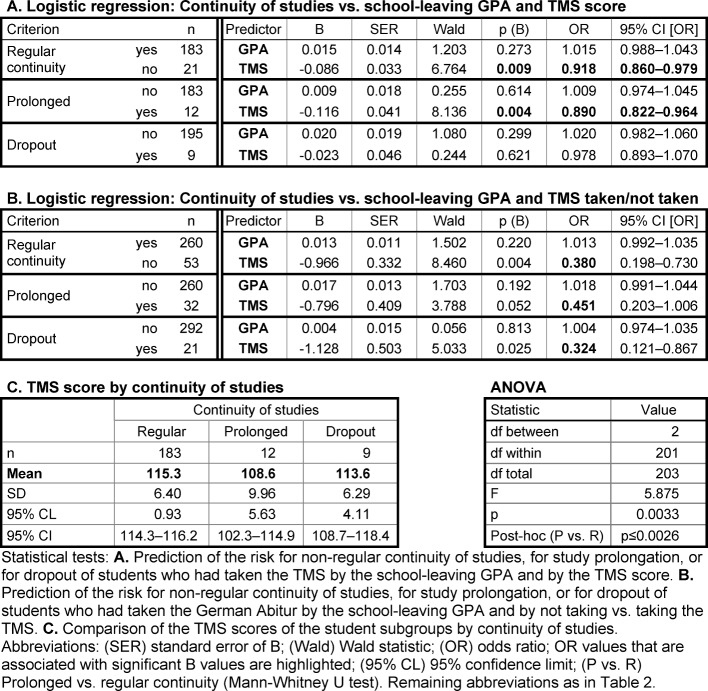
Relationship between continuity of studies, school-leaving GPA, and TMS score among students with the school-leaving grade 1.0

**Table 4:**
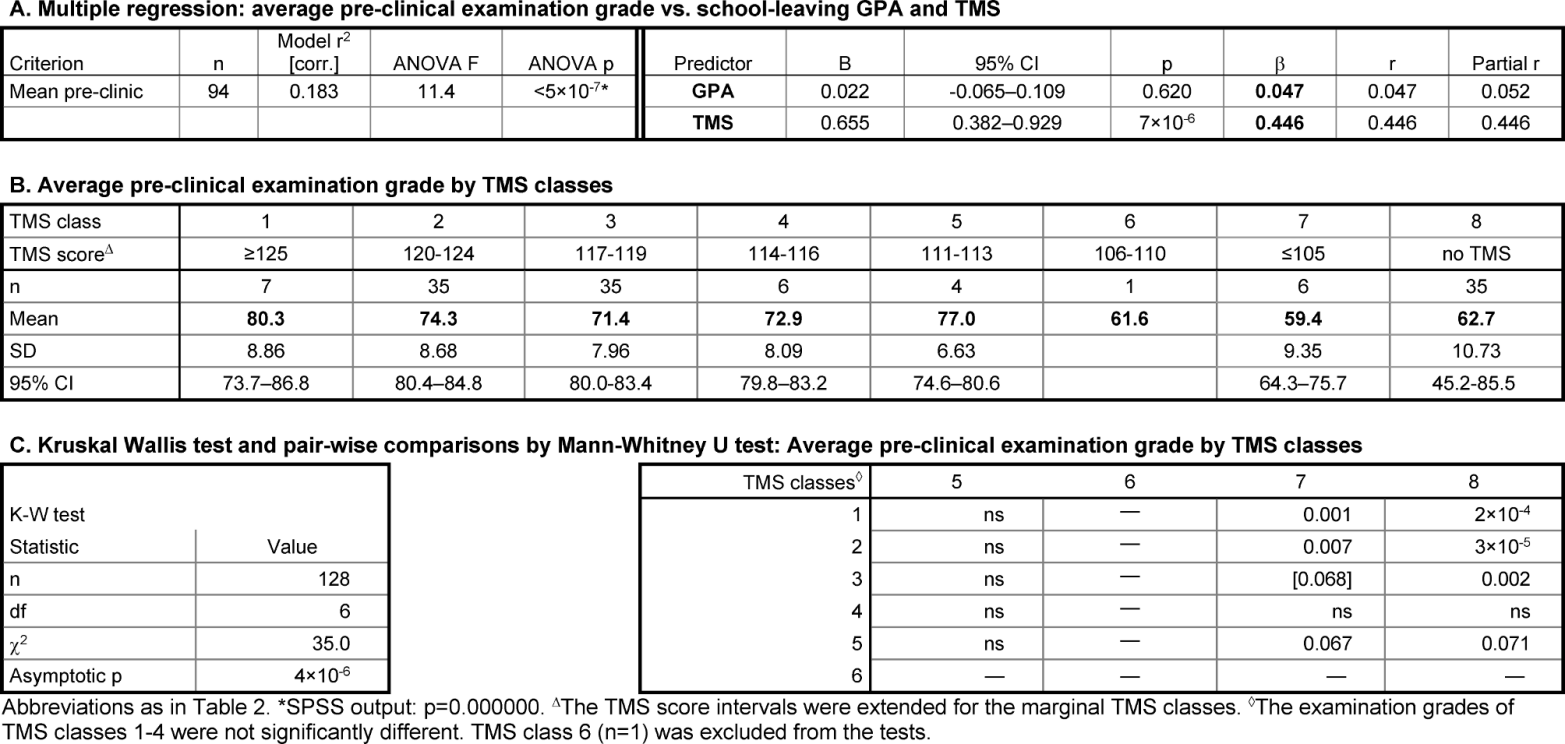
Relationship between academic performance, school-leaving GPA, and TMS score among students with the school-leaving grades 2.0-2.3

**Table 5:**
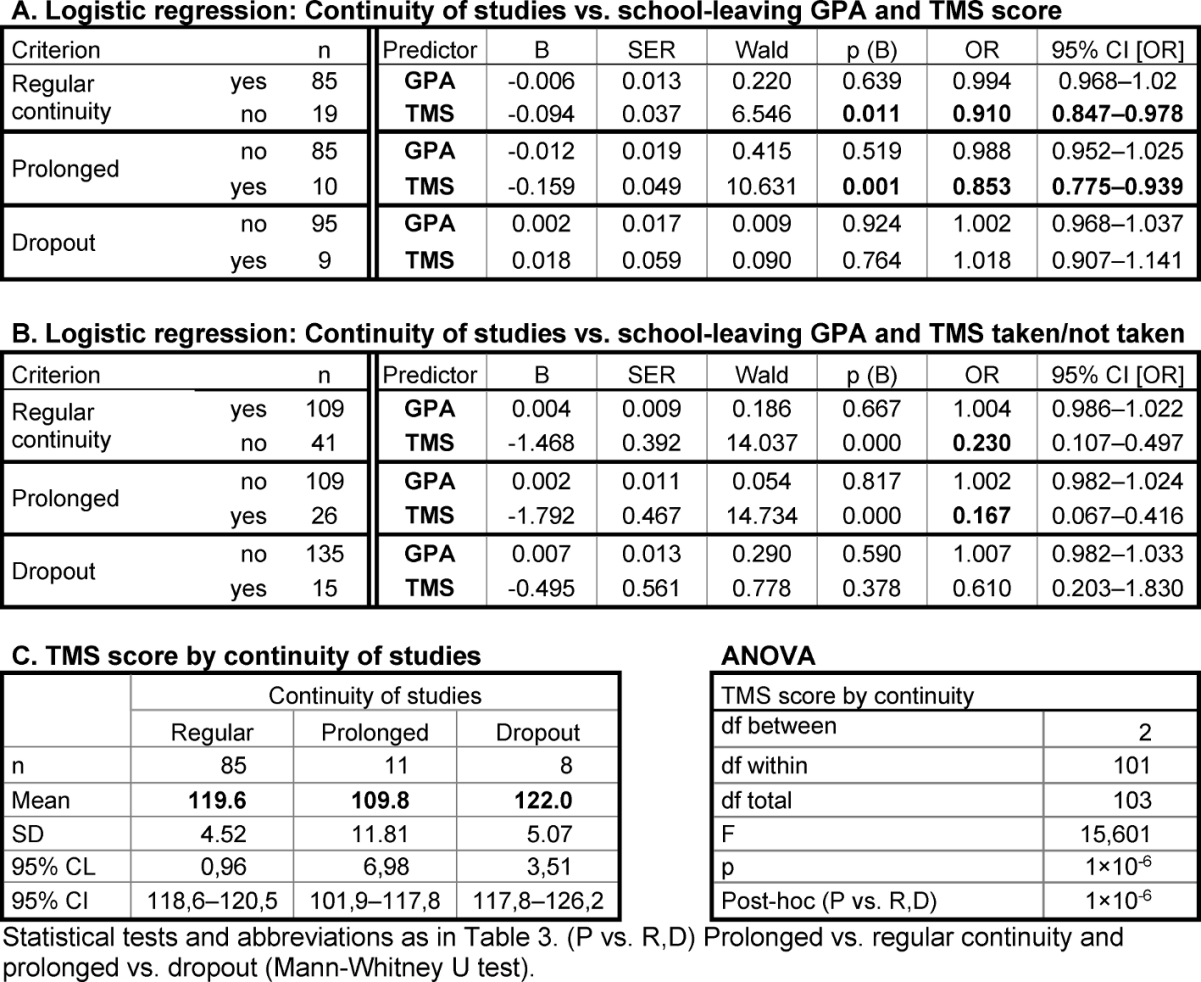
Relationship between continuity of studies, school-leaving GPA, and TMS score among students with the school-leaving grades 2.0-2.3

**Table 6:**
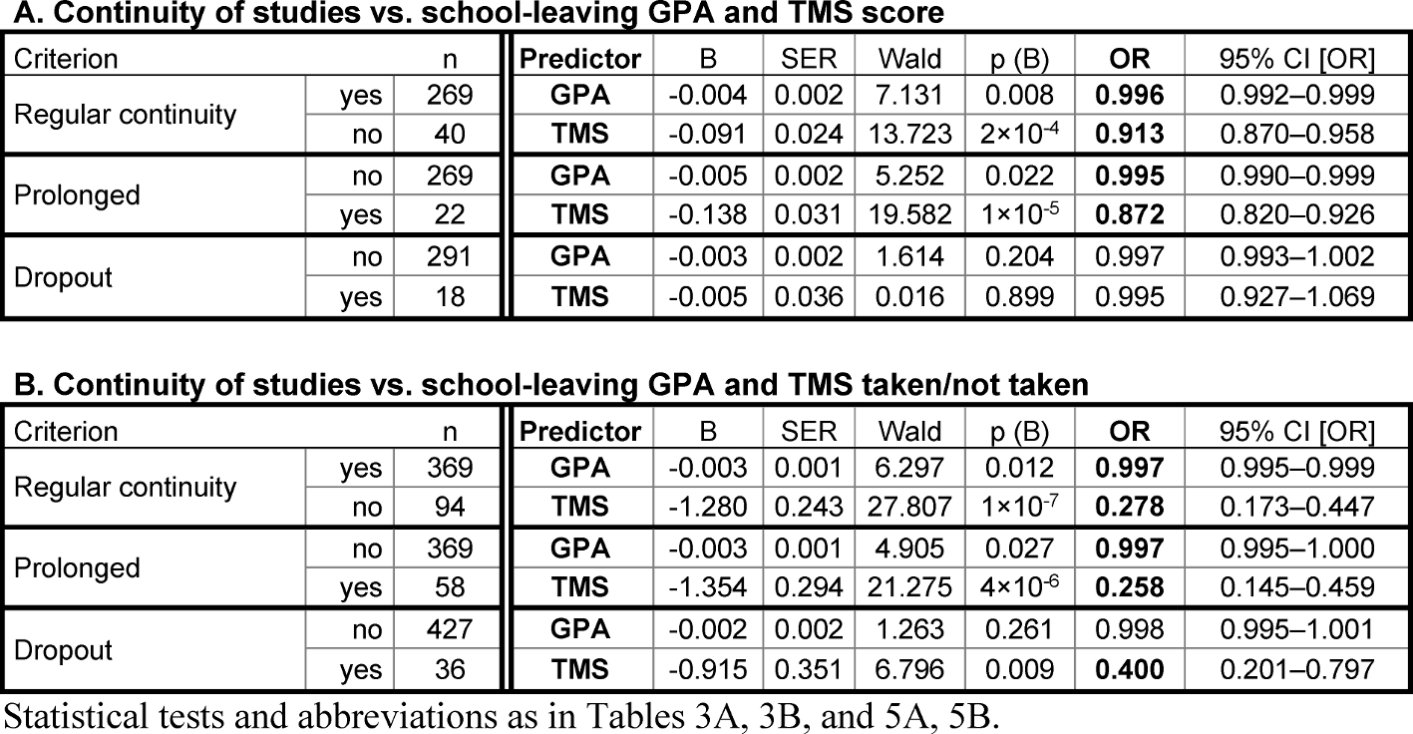
Logistic regression analysis of the relationship between continuity of studies and the school-leaving GPA and the TMS score in the pooled sample of best and mediocre school leavers.
